# A registered report on interruptions during visual search: Examining the effects of perceptual and cognitive load

**DOI:** 10.3758/s13414-026-03360-2

**Published:** 2026-10-28

**Authors:** Emily Seeligmüller, Jeremy M. Wolfe, Jan Philipp Röer

**Affiliations:** 1 Department of Psychology and Psychotherapy, Witten/Herdecke University, Alfred-Herrhausen-Straße 44, Witten, 58455 Germany; 2 Brigham and Women’s Hospital, Boston, MA USA; 3 Harvard Medical School, Boston, MA USA

**Keywords:** Visual search, Interruption, Complexity, Perceptual load, Cognitive load

## Abstract

Interruptions during visual search tasks significantly impair search performance, leading to prolonged reaction time (RT) and reduced accuracy. The interruption’s complexity has been proposed as one important factor that might modulate this decline in performance. However, previous research on interruption complexity during visual search has not clearly differentiated between perceptual and cognitive load, making it difficult to evaluate their relative contributions. This preregistered study examines the effects of interruption complexity on visual search performance by independently manipulating perceptual and cognitive load. Participants performed a visual search task interrupted by a running memory span task. During the interruption task, numerical items were either clearly visible (low perceptual load) or masked with Gaussian visual noise (high perceptual load), and participants recalled either the last item (low cognitive load) or the last four items of the list (high cognitive load). We investigated whether perceptual and cognitive effects are additive and whether one factor is more potent than the other. The results advance theoretical knowledge and contribute to a clearer understanding of the underlying mechanisms of interruption-related performance costs.

Interruptions during visual search are ubiquitous in our everyday lives, and they can significantly affect search performance. There is ample evidence that interruptions during both basic and applied visual search tasks can lead to a substantial decline in performance, increasing reaction time (RT; Alonso et al., 2021; Drew et al., 2018; Radović et al., 2022) and reducing task accuracy (Alonso et al., 2021; Wynn et al., 2018). However, the underlying causes of these effects are presently unclear. Among various contributing factors, the level of interruption complexity has been discussed as a potentially important determinant of the performance decline associated with interruptions (Brazzolotto & Michael, 2019, 2020; Williams & Drew, 2017). In this preregistered study, we will systematically investigate the effects of interruption complexity on visual search performance by orthogonally manipulating perceptual and cognitive load. We hope that the results will provide a more comprehensive understanding of the individual and interactive effects of perceptual and cognitive load.

Visual search is an essential part of various aspects of our lives. We are constantly looking for things (target objects) among other things (distractors)—for example, walking down a crowded aisle of a supermarket, scanning cluttered shelves stacked with many colorful and similar packages, trying to find that one familiar cereal box, or driving through the city, processing street signs, headlights, storefronts, and pedestrians while searching for a parking space. A rich body of literature examines the underlying visual search mechanisms (for reviews, see Chan & Hayward, 2013; Eckstein, 2011; Wolfe, 2020, 2021). Most of these experiments are conducted in a psychological laboratory so that participants can fully concentrate on the focal task. However, this is in stark contrast to the real world, which can be riddled with distractions and interruptions such as push notifications, ringing phones, and advertisements that compete for our attention. Although these interruptions may seem ordinary and often go unnoticed, they can have serious consequences (Balint et al., 2014; Klauer et al., 2014; Williams & Drew, 2017). For instance, interruptions while driving can increase the risk of car accidents (Klauer et al., 2014), and interruptions during a diagnostic process can cause serious medical errors (Balint et al., 2014; Wynn et al., 2018). Different approaches have been employed to study the effects of interruptions across various contexts, including basic laboratory (Alonso et al., 2021; Radović et al., 2022), medical (Balint et al., 2014; Drew et al., 2018), and traffic settings (Borowsky et al., 2016; Klauer et al., 2014; Loft et al., 2015). Across all these settings, interruptions have a negative impact on visual search performance in the form of longer RT and increased error rates (for review, see Cambronero-Delgadillo et al., 2024). Additionally, changes in eye movements have been observed, particularly regarding the duration and frequency of the fixation (Godwin et al., 2013). In interrupted conditions, fixation duration increases and fixation frequency rate decreases compared with uninterrupted conditions. However, interruption effects do not appear to be uniform across all types of search tasks. A recent foraging study by Hong and Wolfe (2024), for example, found that foraging behavior remained relatively resistant to interruptions.

One possible explanation for the commonly observed decline in performance associated with interruptions is that they impair the reactivation and retention of goal-relevant information (Altmann & Trafton, 2002; Hodgetts & Jones, 2006). Visual search is often considered a perceptual task; however, visual search, especially when it gets interrupted, also relies on memory processes, such as maintaining information about the search goal and previously searched areas (Altmann & Trafton, 2002; Brudzinski et al., 2007; Hodgetts & Jones, 2006; Ratwani et al., 2007; Ratwani & Trafton, 2006, 2008). According to the Memory for Goals framework, the activation of goal-relevant representations is influenced by three crucial constraints: the interference level, the strengthening constraint, and the priming constraint (Altmann & Trafton, 2002). Postinterruption performance may therefore be determined by the extent to which these processes are disrupted. Increased interference caused by previously activated but no longer relevant information can disrupt the processing of goal-relevant information making it more difficult to resume the primary search task (Altmann & Trafton, 2002; Alves & Bueno, 2017; Hodgetts & Jones, 2006). In addition to the activation level of goal-relevant information, spatial memory representations play an important role (Brudzinski et al., 2007; Ratwani & Trafton, 2006; 2008). Previous research has shown that spatial representations, such as remembering where the search was stopped and which regions have already been examined, support the resumption of the primary task. Ratwani and Trafton (2008) demonstrated that especially interruption tasks that engage spatial working memory tend to disrupt primary task performance more strongly than nonspatial interruptions.

Many studies have examined not only the consequences of interruptions but also characteristics that could modulate these effects. Specifically, the duration (Lyyra et al., 2012), frequency (Radović et al., 2022), and type of interruption (Borowsky et al., 2016; Drew et al., 2018; Kujala, 2013; Kujala & Saariluoma, 2011) have been identified as critical determinants.

Another proposed key factor is complexity; both the complexity of the primary task (for a review, see Liu & Li, 2012) and, most importantly for the present study, the complexity of the interruption (Brazzolotto & Michael, 2019, 2020; see also Cades et al., 2008). Interruption complexity can substantially affect primary task performance across many different tasks (Gillie & Broadbent, 1989; Hodgetts & Jones, 2006; Kapitsa & Blinnikova, 2003; Nakajima & Sato, 1989; Radović & Manzey, 2022). For example, in a procedural task with sequential constraints, more complex interruptions led to greater impairments in postinterruption performance, reflected in longer resumption times and increased sequence errors compared with simpler interruptions (Radović & Manzey, 2022). Similarly, Fischer and Glanzer (1986) demonstrated that participants took longer to read the first sentence after a demanding interruption in a text comprehension study. Perceptual and cognitive factors play an important role in these interruption complexity effects (Coleman & Elliott, 2021; Fisher et al., 2018; Klemen et al., 2009; Macdonald & Lavie, 2011; Murphy & Greene, 2017).

Lavie’s “Load theory” is relevant to how we understand the role of interruption (Lavie, 1995; Lavie & Tsal, 1994; Lavie et al., 2004). Load theory states that high perceptual load enhances selective attention. When perceptual capacity is fully occupied, irrelevant stimuli are less likely to be processed. As a result, distracting stimuli can be ignored more effectively, leading to improved performance (Forster & Lavie, 2009; Lavie, 1995). Translated to the present study, if it is an interruption with high perceptual load, the “irrelevant” stimuli that are less likely to be processed may include the interrupted, primary task. Conversely, a high cognitive load depletes limited cognitive resources essential for higher cognitive processes (Lavie & De Fockert, 2005). Once these resources are entirely employed, there is not enough capacity left to inhibit distracting stimuli. This depletion can affect task performance by reducing the ability to ignore irrelevant stimuli (De Fockert et al., 2001; Lavie & De Fockert, 2005; Murphy & Greene, 2017). Studies have supported these theories by showing that perceptual load reduces the processing of irrelevant stimuli, whereas cognitive load increases distractor interference (Lavie et al., 2004; Murphy & Greene, 2017; but see Fisher et al., 2018; Klemen et al., 2009). Again, however, note that in most of the experiments, the demands of the primary task were manipulated (i.e., perceptual and cognitive load), and not the task demands of the interruption.

From the perspective of the Memory for Goals theory, load manipulation may affect distinct aspects of goal processing. High perceptual load may limit the reactivation of goal-relevant and spatial information, as attentional resources are fully occupied by the interruption task, limiting opportunities for rehearsal and refreshing aspects of the primary task. Cognitive load during an interruption may interfere with the sustaining of goal-relevant information, as both rely on limited working memory resources (Altmann & Trafton, 2002; Lavie & De Fockert, 2005). This may lead to reduced goal memory and accessibility of goal-relevant representations after the interruption. Consequently, perceptual and cognitive load manipulation during an interruption may differentially influence goal maintenance, decay and reactivation, leading to decreased postinterruption performance.

The role of interruption load has not been extensively investigated. Brazzolotto and Michael (2019, 2020) demonstrated that high interruption complexity delayed the resumption of visual search tasks. Williams and Drew (2017), by contrast, found no effect of interruption complexity. A potential explanation for this inconsistency could be the lack of a clear distinction between perceptual and cognitive load. The few studies that have focused on manipulating interruption complexity during visual search have not systematically differentiated between these two factors. Instead, in these studies, interruptions were rather heterogeneous and often either low or high in both perceptual and cognitive load, making it difficult to assess their relative contributions. Therefore, we do not know which factor is more important or whether the two effects are additive, or not.

One of the main goals of this preregistered study was to close this knowledge gap. To this end, we orthogonally manipulated perceptual and cognitive load in a within-subject design using a large sample to ensure sufficient statistical power. Since this was the first study to systematically investigate the perceptual and cognitive effects of interruption complexity during visual search, we used established methods and a straightforward design. For a search task, we used a classic letter search task (e.g., Godwin et al., 2013; Radović et al., 2022; Wolfe, 2020), while the interruption task contained a running memory span task (cf. Brazzolotto & Michael, 2019, 2020; see also Pollack et al., 1959). We manipulated perceptual load by varying the encoding difficulty by adding Gaussian visual noise to the memory span items (cf. Hughes et al., 2013; see also Marsh et al., 2020). A slightly novel approach we applied was to manipulate cognitive load by varying the number of to-be-recalled memory span items. This resulted in a 2 × 2 design: (1) Low perceptual load and low cognitive load, (2) low perceptual load and high cognitive load, (3) high perceptual load and low cognitive load, and (4) high perceptual load and high cognitive load.

The following explicit and testable hypotheses can be formulated on the basis of the above theoretical considerations: (H1) Interrupted visual search trials will result in longer total search time and lower search accuracy compared with uninterrupted trials. (H2) High perceptual load during the interruption will impair visual search performance, resulting in longer total search time, longer resumption time, and lower search accuracy compared with the low perceptual load condition. (H3) High cognitive load during the interruption will impair visual search performance, resulting in longer total search time, longer resumption time, and lower search accuracy compared with the low cognitive load condition. A priori, no clear hypotheses were formulated regarding the interaction of perceptual and cognitive load, but we tested whether their effects were additive or interactive.

## Methods

### Power analysis

We will collect data from 84 participants. If we have fewer than 84 usable datasets by the end of the testing phase, we will conduct additional testing sessions to reach the target sample size. The main research question was whether perceptual and cognitive complexity modulate the effects of interruptions on visual search performance. Therefore, the critical test was the comparison of visual search performance under low-load and high-load conditions. A power analysis was conducted with G*Power (Faul et al., 2007) to detect the main effects of perceptual and cognitive load. It used probabilities for Type I and Type II errors of α = β = 0.05 and an assumed correlation of ρ = 0.5 among the levels of the repeated measures variable. The analysis concluded that a sample size of *N* = 84 would allow for the detection of an effect size of* f* = 0.2, which corresponds to a small [*f* = 0.1] to medium [*f* = 0.25] effect according to Cohen (2013).

### Participants

The sample consisted primarily of students at Witten/Herdecke University, who participated in return for course credit. All participants were between 18 and 49 years old and reported normal or corrected-to-normal vision.

### Apparatus and materials

**Fig. 1:**
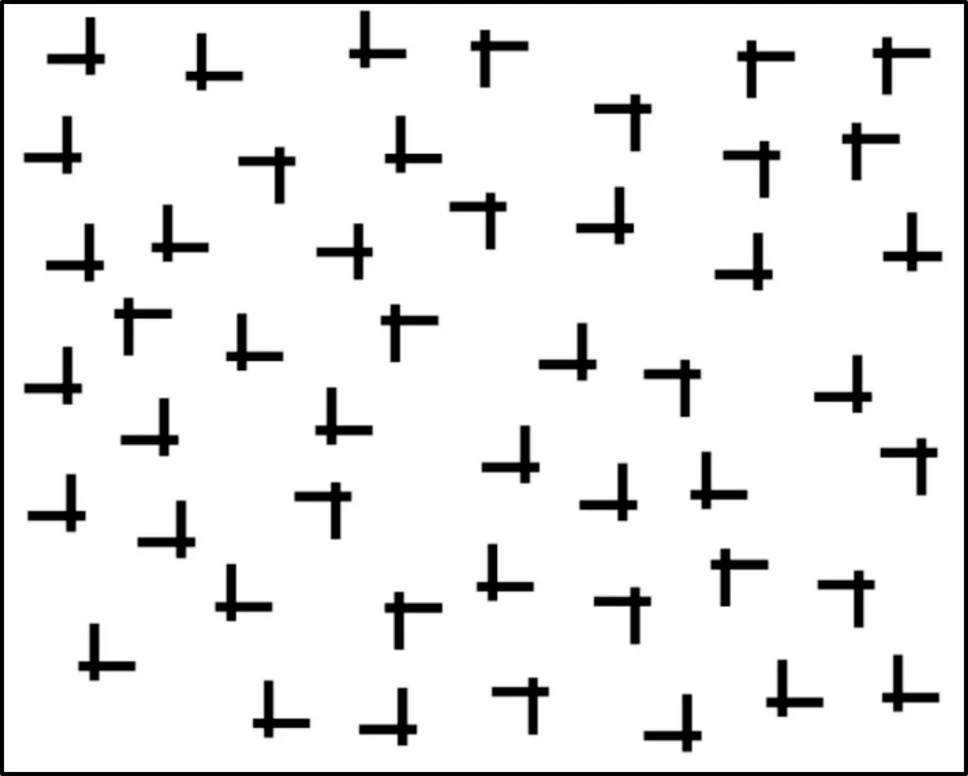
Illustration of a target-present trial with 48 items. The task for the participants is to search for the *T*-shaped target

All stimuli were presented on a 24-in. screen with a resolution of 1,920 × 1,080 and a refresh rate of 50–60 Hz. The primary task involved a visual letter search (*T* among *L*; Godwin et al., 2013; Horowitz & Wolfe, 1998; Lleras et al., 2005). Each trial displayed one of two set sizes (48, 72), containing either zero, one, two, or four *T*-shaped targets (target count), with the remaining items consisting of *L*-shaped distractors. The horizontal bar of the letters was displaced along the vertical stem so that it intersected the stem, creating a slight variation in appearance. Stimuli were distributed evenly without overlaps rotated to one of four angles (0°, 90°, 180°, or 270°), as illustrated in Fig. 1.

**Fig. 2:**
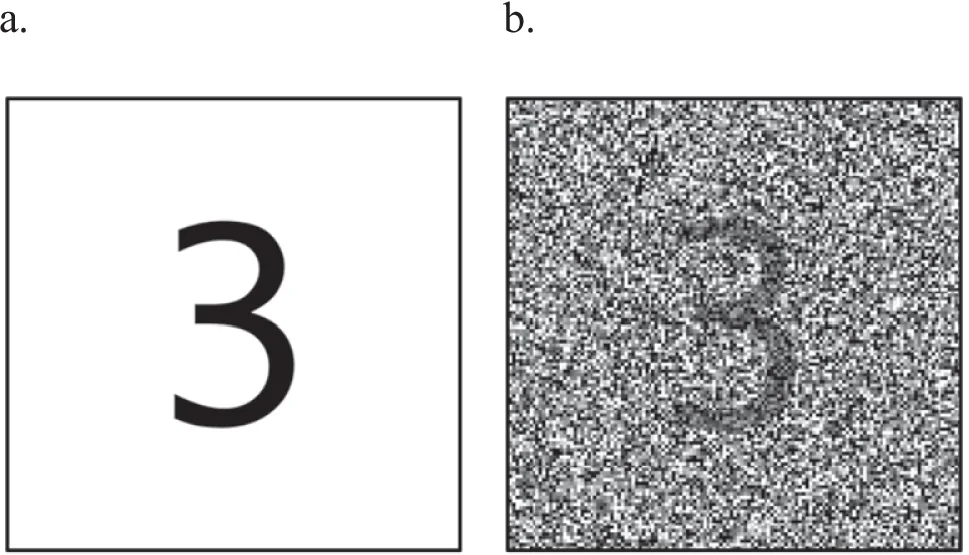
Illustration of the low perceptual load (**a**) and high perceptual load condition (**b**)

During each interruption, participants performed a running memory span task (Pollack et al., 1959). A series of five, six, seven, or eight items were sampled randomly without replacement from the set [0, 1, … 9]. These items were presented consecutively at a rate of 500 ms on and 250 ms off in 72-point Arial black font in the center of the screen. They appeared either on a white background or masked by static Gaussian visual noise (400%). In the masked condition, item transparency was set to 50% (see Fig. 2). Within each individual task, all items were of equal size and the viewing distance of 45 cm was maintained using a chin rest, ensuring that the vertical and horizontal angles remained constant.

### Procedure

Data collection was conducted in a single session lasting approximately 60 min using a within-subject design. Participants were seated in a room with minimal environmental distractions and received written instructions. Their primary task consisted of a visual search task. Participants were instructed to click exactly once on every target displayed or to refrain from clicking on items in target-absent trials. Participants indicated completion of the search by clicking a “Continue” button using the computer keyboard.

In 50% of the trials, an interruption occurred during the search in the form of a running memory span task. Interruption onset was either 4,000 ms or 8,000 ms to limit any anticipatory effects. Interruption duration was standardized across all conditions (15 s), starting with a display of placeholders that marked the input fields in which the participants would later have to enter the digits. The number of placeholders informed participants about the number of digits they were required to memorize and remained visible throughout the digit presentation. Following the presentation of the items, participants were prompted to recall the digits using the number block of the keyboard. During the interruption, a progress bar was displayed, indicating the time remaining until the visual search task resumed. If participants entered all digits before the 15-s interruption period had passed, the response interface remained visible until the progress bar reached the end of the interruption period.

The numerical items were either clearly visible (low perceptual load; Fig. 2a) or masked with Gaussian visual noise (high perceptual load; Fig. 2b), and participants were required to recall the last item (low cognitive load) or the last four items (high cognitive load) of the list. Trials were presented in random order, with equal numbers of trials for both set size and target count across the experiment, and interruption onset balanced across the interrupted trials. The experiment consisted of a total of 128 trials (64 uninterrupted and 16 interrupted trials in each of the four load–condition combinations).

To familiarize themselves with the task and instructions, participants completed eight training trials, consisting of four uninterrupted and four interrupted trials, prior to the experiment proper.

### Preregistered analyses

Prior to the analyses, statistical assumptions were tested and any violations reported. In cases where the sphericity assumption of an ANOVA was compromised, the Greenhouse–Geisser correction was applied. For all significant ANOVAs, pairwise post hoc comparisons were conducted using the Bonferroni correction to control for multiple comparisons. The significance level (α) for all analyses was set to .05. RT and accuracy were the main dependent variables. Three different RT measures were collected: RT1 was defined as the total search time (i.e., the interval from the beginning to the end of the search in uninterrupted trials, and the same interval with the interruption duration subtracted in interrupted trials). Unless otherwise specified, RT1 was used as the dependent variable for all RT-related analyses. RT2 was defined as the time from the reappearance of the visual search display after the interruption until search completion, and RT3 was defined as the time until the first goal-related action after the interruption. We excluded trials with RT1s below 200 ms and those exceeding 2.5 standard deviations from the participant’s mean prior to further analyses. The analysis of accuracy was based on arcsine–square–root-transformed error rates. There were three error categories: omission errors (failure to detect a target), repetition errors (repeated selection of an already identified target), and misidentification errors (selection of a nontarget). A trial was considered correct if none of these errors occurred and incorrect if one or more errors occurred.

#### Confirmatory analyses

##### Interruption

To examine interruption effects on visual search performance, we compared interrupted and uninterrupted trials using paired *t* tests. We expected that interruptions negatively affect visual search performance, indicated by slower RT1 and reduced accuracy. To examine interruption complexity effects on visual search performance, we conducted 2 × 2 repeated-measures ANOVAs with perceptual load (low, high) and cognitive load (low, high) as independent variables. Main effects of perceptual and cognitive load, and an interaction of both factors would be evidence for an effect of interruption complexity on visual search performance. Additionally, we investigated whether the resumption time (RT3) differed across the load conditions using a 2 × 2 repeated-measures ANOVA with perceptual (low, high) and cognitive load (low, high) as within-subject factors and RT3 as the dependent variable.

#### Exploratory analyses

##### Across-trial performance

In exploratory analyses, we examined visual search performance over the course of the experiment. It is possible that participants may adapt to the occurrence of interruptions with repeated presentations due to practice or habituation. To test potential across-trial performance effects, all trials were divided into four successive blocks, each consisting of 32 trials. Performance on interrupted and uninterrupted trials was compared across these blocks using 2 × 4 repeated-measures ANOVAs with trial type (interrupted, uninterrupted) and block (1 to 4) as within-subject factors. An interaction of trial type and block in the form of a progressively smaller interruption effect over the course of the experiment would be evidence that participants adapt to interruptions due to practice or habituation.

##### Interruption onset

To examine interruption onset effects, we conducted paired *t* tests comparing performance between trials with interruption onsets at 4,000 ms and 8,000 ms.

##### Primary task difficulty

To investigate the effect of set size on search performance, we calculated search slopes. Search slopes were defined as the difference in mean RT1s between set sizes divided by the difference in the number of items. We used 2 × 2 repeated-measures ANOVAs with set size (48, 72) and trial type (interrupted, uninterrupted) as within-subject factors to examine whether the effect of set size on search performance was different for interrupted and uninterrupted trials. To further examine how interruption complexity interacts with primary task difficulty, we conducted a 2 × 2 × 2 repeated-measures ANOVA with perceptual load (low, high), cognitive load (low, high), and set size (48, 72) as within-subject factors. The same analysis was conducted with target count (zero, one, two, four) as the third within-subject factor.

##### Resumption

In addition, we analyzed whether there was a significant difference between the RT2 in interrupted trials and the RT1 in uninterrupted trials, by using a paired *t* test. The absence of a significant difference might indicate that participants restarted their search from the beginning after the interruption rather than continuing it from the point they left off.

##### Running memory span

To investigate participants’ performance in the interruption task, we conducted a 2 × 2 repeated-measures ANOVA with cognitive (low, high) and perceptual load (low, high) as within-subject factors. The dependent variable was running memory span performance. Furthermore, we investigated whether performance in the running memory span task and in the visual search task was related using correlational analyses.

## Data Availability

After data collection, stimulus material, raw data and analysis code were uploaded and made publicly available at the project page on the OSF (https://osf.io/nketz/).

## Declarations

### Preregistration

We confirm that no data had been collected for the proposed project at Stage 1 submission. After acceptance of the Stage 1 manuscript, the study protocol was publicly preregistered on the Open Science Framework (OSF) project page before data collection began.
